# The Presence of Plant-Associated Bacteria Alters Responses to *N*-acyl Homoserine Lactone Quorum Sensing Signals that Modulate Nodulation in *Medicago Truncatula*

**DOI:** 10.3390/plants9060777

**Published:** 2020-06-22

**Authors:** Debora F. Veliz-Vallejos, Akitomo Kawasaki, Ulrike Mathesius

**Affiliations:** 1 Division of Plant Sciences, Research School of Biology, Canberra, ACT 2601, Australia; apis.dvv@gmail.com (D.F.V.-V.); akitomo.kawasaki@gmail.com (A.K.); 2 CSIRO Agriculture and Food, Canberra, ACT 2601, Australia

**Keywords:** acyl-homoserine lactones, legume, microbiome, nodulation, rhizobia, quorum sensing

## Abstract

Bacteria use quorum sensing signaling for cell-to-cell communication, which is also important for their interactions with plant hosts. Quorum sensing via *N*-acyl-homoserine lactones (AHLs) is important for successful symbioses between legumes and nitrogen-fixing rhizobia. Previous studies have shown that plant hosts can recognize and respond to AHLs. Here, we tested whether the response of the model legume *Medicago truncatula* to AHLs from its symbiont and other bacteria could be modulated by the abundance and composition of plant-associated microbial communities. Temporary antibiotic treatment of the seeds removed the majority of bacterial taxa associated with *M. truncatula* roots and significantly altered the effect of AHLs on nodule numbers, but lateral root density, biomass, and root length responses were much less affected. The AHL 3-oxo-C_14_-HSL (homoserine lactone) specifically increased nodule numbers but only after the treatment of seeds with antibiotics. This increase was associated with increased expression of the early nodulation genes *RIP1* and *ENOD11* at 24 h after infection. A 454 pyrosequencing analysis of the plant-associated bacteria showed that antibiotic treatment had the biggest effect on bacterial community composition. However, we also found distinct effects of 3-oxo-C_14_-HSL on the abundance of specific bacterial taxa. Our results revealed a complex interaction between plants and their associated microbiome that could modify plant responses to AHLs.

## 1. Introduction

Plant-associated bacteria can have beneficial effects on plant performance, for example, by altering plant growth and development or by inducing tolerance to diverse biotic and abiotic stresses [1,2,3]. Bacterial and fungal species can colonize all parts of the plant, including surfaces and the apoplast of roots, shoots, and seeds. Some bacteria also infect plants intracellularly, most prominently nitrogen-fixing bacteria that invade the inside of root hairs and cortical cells, although they remain outside the plant plasma membrane [4]. Plants can be colonized by various bacteria during different developmental stages and can also inherit some of their bacterial consortia via seeds in a process called vertical transmission. The seed-borne bacterial community structure is able to disperse systemically throughout the plant, colonizing roots and nodules [5,6].

Many plant-associated bacteria use cell-to-cell signaling via quorum sensing signals to coordinate behaviors that support successful colonization or invasion of plant hosts [7,8]. Gram-negative bacteria typically use acyl-homoserine lactone (AHL) signals, which control behaviors important for plant-microbe interactions, such as biofilm formation, bacterial motility, expression of pathogenicity genes, plasmid transfer, production of antibiotics, expression of genes required for successful symbiotic nitrogen fixation, and many others, in a density-dependent manner [9]. A possible advantage for controlling these behaviors in a density-dependent manner is that bacterial populations can build up to effective numbers before triggering plant defense responses [10,11,12].

Plants can detect the presence of AHLs and respond in a number of specific ways, even to very low (pM) concentrations of AHLs [13]. It has been shown that exposure of plants to AHLs can elicit a wide range of molecular and physiological responses in plants that are relevant for the outcome of plant–microbe interactions. One of the responses of plants to AHLs includes the production of quorum sensing mimic compounds, which can interfere with bacterial quorum sensing [13,14,15]. Other responses include changes in defense and developmental pathways in plants. AHLs can interfere with plant growth and development by altering plant hormone and signaling pathways, e.g., [16,17,18,19,20,21]. In addition, AHLs mediate plant defense responses and can often confer enhanced tolerance to pathogens, e.g., [22,23,24,25,26,27].

In legumes, which are colonized by nitrogen-fixing rhizobia, exposure to AHLs can influence the outcome of the symbiosis. During the legume-rhizobia symbiosis, rhizobia are initially present in the soil and are attracted to flavonoid exudates of host roots [28]. Flavonoids activate the synthesis of rhizobial nodulation (Nod) factors, which are perceived by the plant host and trigger a signaling cascade necessary for the invasion of rhizobia into the root and the initiation of nodule development [29,30,31]. In addition to Nod factors, exopolysaccharides are necessary for the successful invasion of rhizobia into root hairs and later inside nodules [32].

AHLs synthesized by rhizobia can influence the outcome of symbiosis by regulating bacterial attachment to root surfaces, host invasion via exopolysaccharide synthesis, plasmid transfer, movement, and expression of nitrogen-fixation genes [33,34,35,36,37,38,39,40]. On the plant side, the perception of rhizobial AHLs by legume hosts is also likely to be important for fine-tuning the symbiosis. Exposure of legumes to AHLs improved nodulation in the model legume *Medicago truncatula*, but this was observed after exposure to the symbiont-produced AHL, 3-oxo-C_14_-HSL (3-oxo-C_14_-homoserine lactone) in one study [41], while a smaller increase in nodule numbers was observed in a different study, which also showed that specific AHLs, including 3-oxo-C_14_-HSL, accelerated the rate of nodulation in *M. truncatula* [42]. The mechanism of the nodulation response remains unknown.

Because legume tissues are concurrently colonized by rhizobia, as well as a multitude of other microbes [43,44], it is possible that quorum sensing signals from these different species cause a range of distinct and overlapping responses in the host plant. It is also likely that plant hosts perceive signals from multiple bacteria at once, and this could influence the overall responses to their specific symbionts. Here, we tested whether the presence of naturally-occurring bacteria associated with the plant host would alter the response of *M. truncatula* to AHLs and how this influenced the plant symbiosis with its specific symbiont, *Sinorhizobium meliloti*. We decided to test this by comparing responses to AHLs in plants from surface-sterilized seeds in comparison with seeds receiving an additional antibiotic treatment that was able to remove the majority of the bacterial taxa associated with the plant. This was done to make sure that all the bacteria that were present were naturally associated with the plant and well able to colonize the plant. The seeds used in our study originated from field-grown *M. truncatula* plants that we observed to harbor a variety of plant-associated bacteria.

## 2. Results

### 2.1. The Positive Effect of 3-oxo-C_14_-HSL on Nodule Numbers Depended on the Presence of Plant-Associated Bacteria

**Figure 1:**
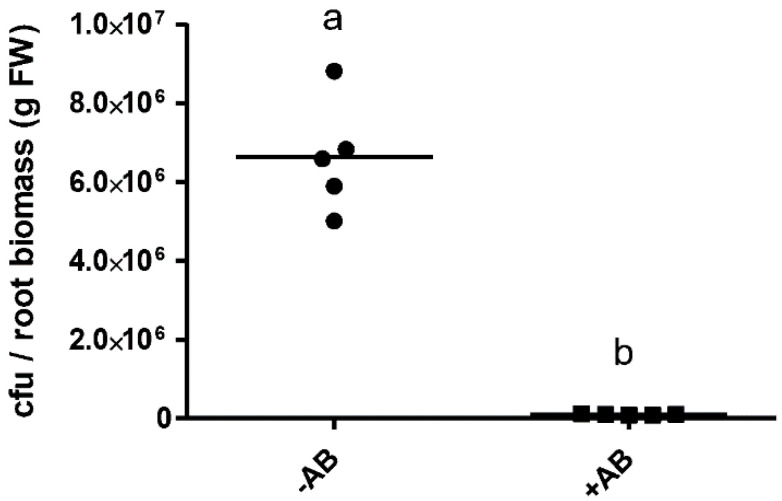
Effect of a 6 h antibiotic treatment of *M. truncatula* seeds on root-associated bacteria four days after germination. Bacterial abundance was estimated from culturable bacterial colonies and was normalized against the root biomass of each plant. Significant differences between the treatments are indicated with different letters; *p* < 0.05 (Student’s *t*-test). Abbreviations: cfu: colony-forming units; FW: fresh weight. For *M. truncatula* roots of this age, 1 g of fresh weight is equivalent to about 50 roots; thus, each root (approximately 4 cm long) in the −AB/+AB treatment groups would harbor on average around 120,000/2000 colony-forming units, respectively.

We initially wanted to confirm the specific effect of 3-oxo-C_14_-HSL on nodulation in *M. truncatula* in the presence and absence of a short antibiotic (AB) treatment at the seed sterilization stage. To test the effectiveness of the AB treatment, we quantified the number of culturable bacteria from ground roots. The AB treatment significantly reduced the number of culturable bacteria in four-day-old seedlings, from about 120,000 culturable cells per root in the –AB-treated plants to around 2000 in the +AB treated plants (Figure 1). Despite these numbers, even the non-AB treated plants, originating from seeds that were treated with 6% sodium hypochlorite before germination, same as the AB-treated seeds, did not look contaminated, nor did we see any obvious colonies growing on or around the seedlings that were growing on Fåhraeus agar plates (data not shown).

**Figure 2:**
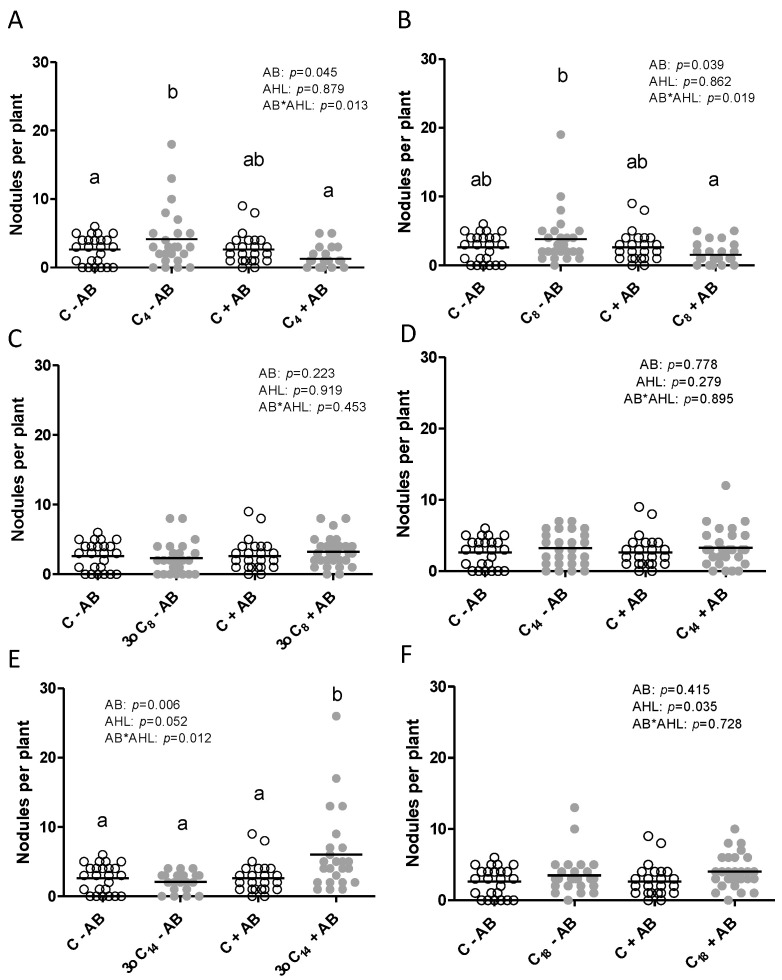
Nodule numbers of *M. truncatula* plants exposed to 1 μM AHLs (acyl-homoserine lactone) and inoculated with rhizobia, measured at 21 days after inoculation. Plants were treated with (grey) or without (white) (**A**) C_4_-HSL, (**B**) C_8_-HSL, (**C**) 3-oxo-C_8_-HSL, (**D**) C_14_-HSL, (**E**) 3-oxo-C_14_-HSL, or (**F**) C_18_-HSL. The control treatments (‘C’) included the same dilution of the solvent as the AHL treatments. Different letters indicate significant differences between the treatments when the interaction is significant (Restricted Maximum Likelihood test *p* < 0.05). No post hoc test when the interaction is not significant. AB: antibiotic treatment.

As shown in Figure 2, the presence of 3-oxo-C_14_-HSL at 1 μM concentration in the plant growth medium increased nodule numbers significantly compared to the solvent control. The concentration of 1 μM chosen here compared with concentrations of AHLs was found to be biologically active in other studies and species [13,16,17,20,42] and was within the range of realistic AHL concentrations in the rhizosphere [23].

However, the positive effect of 3-oxo-C_14_-HSL on nodule numbers was only found when seeds had been treated with AB, suggesting that the presence of bacteria inside the seedlings, most likely derived from the seed, affected this response. We also tested five other AHLs to examine whether the dependence of AHL effects on plant-associated bacteria was AHL structure-dependent. We chose the short-chain AHLs—C_4_-HSL and C_8_-HSL—which are produced by *Pseudomonas aeruginosa* [45] and *Sinorhizobium meliloti* [46], respectively, and which both had a negative effect on nodulation in our previous study that had been conducted with the same AB treatment used here [41]. In addition, we chose 3-oxo-C_8_-HSL and C_14_-HSL to test the effect of the 3-oxo group on AHL-dependent plant phenotypes. Both of those AHLs are also produced by *S. meliloti* [14]. In addition, we chose one long-chain AHL, C_18_-HSL, which is also produced by *S. meliloti* [46]. Exposure of *M. truncatula* to C_4_-HSL and C_8_-HSL showed a clear interaction between AB treatment and AHL treatment, with a significantly lower nodule number in the AHL + AB treatment compared to the AHL–AB treatment (Figure 2). Exposure to 3-oxo-C_8_-HSL or C_14_-HSL did not affect nodule numbers. Exposure to C_18_-HSL slightly but significantly increased nodule numbers in both +AB and −AB-treated plants, with no interaction between AHL and AB treatment (Figure 2).

**Figure 3:**
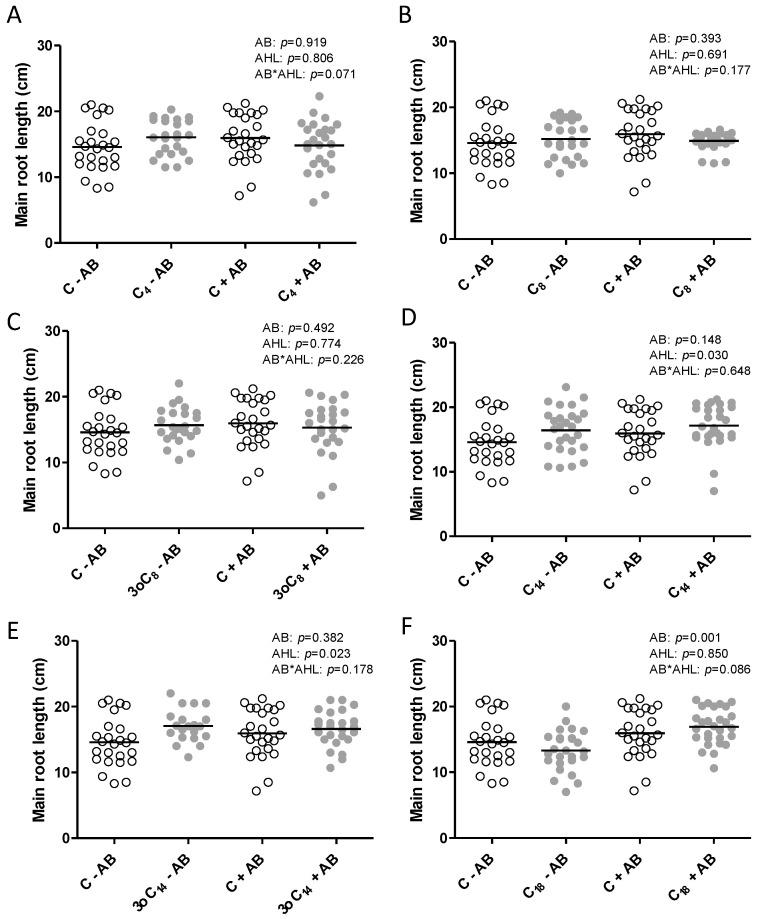
Root length of *M. truncatula* plants exposed to 1 μM AHLs and inoculated with rhizobia, measured at 21 days after inoculation. Plants were treated with (grey) or without (white) (**A**) C_4_-HSL, (**B**) C_8_-HSL, (**C**) 3-oxo-C_8_-HSL, (**D**) C_14_-HSL, (**E**) 3-oxo-C_14_-HSL, or (**F**) C_18_-HSL. The control treatments (‘C’) included the same dilution of the solvent as the AHL treatments. Different letters indicate significant differences between the treatments when the interaction is significant (REML test *p* < 0.05). No post hoc test when the interaction is not significant. AB: antibiotic treatment.

We also measured other root traits and found that both C_14_-HSL and 3-oxo-C_14_-HSL had a small but statistically significant positive effect on the main root length, while the AB treatment did not affect root length, except in the C_18_-HSL treatment (Figure 3). The short-chain AHLs did not affect root length in the +AB or the −AB treatment (Figure 3).

**Figure 4:**
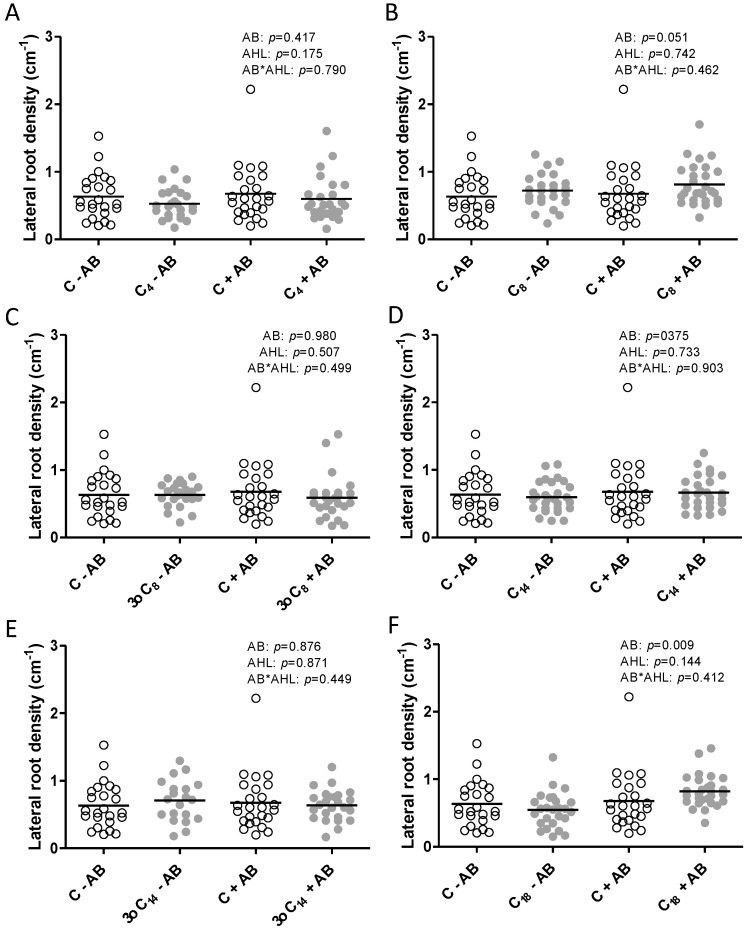
Lateral root density (number of primary lateral roots/cm main root length) of *M. truncatula* plants exposed to 1 μM AHLs and inoculated with rhizobia, measured at 21 days after inoculation. Plants were treated with (grey) or without (white) (**A**) C_4_-HSL, (**B**) C_8_-HSL, (**C**) 3-oxo-C_8_-HSL, (**D**) C_14_-HSL, (**E**) 3-oxo-C_14_-HSL, or (**F**) C_18_-HSL. The control treatments (‘C’) included the same dilution of the solvent as the AHL treatments. Different letters indicate significant differences between the treatments when the interaction is significant (REML test *p* < 0.05). No post hoc test when the interaction is not significant. AB: antibiotic treatment.

Lateral root density was not affected by any of the AHLs, with a minor effect of the AB treatment in the C_18_-HSL-treated plants (Figure 4).

**Figure 5:**
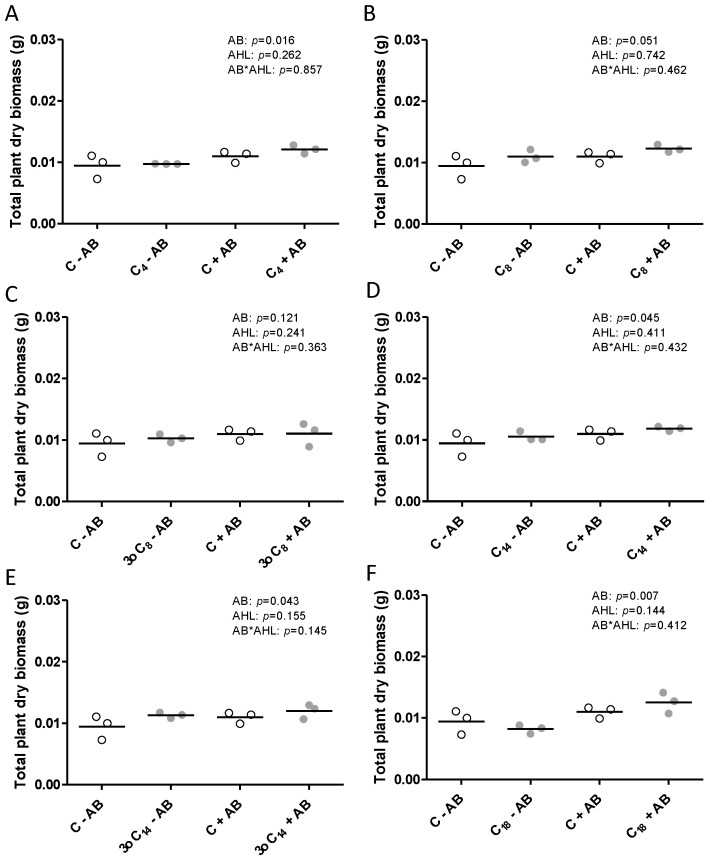
Total dry biomass (root and shoot biomass) (g plant^−1^) in *M. truncatula* plants exposed to 1 μM AHLs and inoculated with rhizobia, measured at 21 days after inoculation. Plants were treated with (grey) or without (white) (**A**) C_4_-HSL, (**B**) C_8_-HSL, (**C**) 3-oxo-C_8_-HSL, (**D**) C_14_-HSL, (**E**) 3-oxo-C_14_-HSL, or (**F**) C_18_-HSL. The control treatments (‘C’) included the same dilution of the solvent as the AHL treatments. Different letters indicate significant differences between the treatments when the interaction is significant (REML test *p* < 0.05). No post hoc test when the interaction is not significant. AB: antibiotic treatment. Each one of the three data points represents a measurement of 10 roots combined, because individual roots were too light to weigh accurately.

Plant dry biomass was slightly but significantly increased by the antibiotic treatment irrespective of the AHL treatment for C_4_-, C_14_-, 3-oxo-C_14_-, and C_18_-HSL-treated plants (Figure 5).

Overall, these results demonstrated that the positive effect of 3-oxo-C_14_-HSL on nodulation was specific and independent of other root phenotypes, while negative effects of the short-chain AHLs on nodule numbers were also dependent on the presence of root-associated bacteria, but in that case, the –AB-treated seedlings were more affected than the +AB-treated seedlings. In addition, we found that the presence of the 3-oxo group had different effects on 3-oxo-C_8_-HSL and 3-oxo-C_14_-HSL. Overall, the interaction between AHL structure and its effect on nodulation was in some, but not all cases, dependent on the microbial abundance and composition of the roots (further examined below).

**Figure 6:**
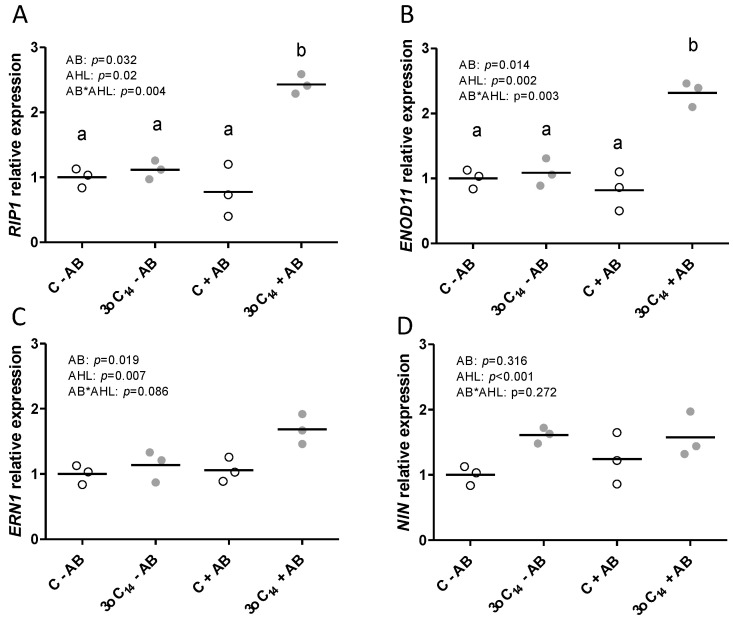
Effect of AHLs on the relative quantification of gene expression of four-day-old *M. truncatula* roots 24 h after rhizobia inoculation treated with 1 µM 3-oxo-C_14_-HL (‘3oC14’; grey) or solvent control (‘C’, white). (**A**) *RIP1*, (**B**) *ENOD11*, (**C**) *ERN1*, (**D**) *NIN1.* All expression ratios are relative to the control (‘C’)-treated samples (no AHL, no AB) and normalized against *GAPDH* expression. REML test *p* < 0.05 with Bonferroni’s post-test. No post hoc test when the interaction is not significant. Each sample contained five roots per biological replicate.

To investigate at which stage of nodulation the AHL 3-oxo-C_14_-HSL started to have a positive effect, we quantified the expression of four nodulation genes that are known to be induced during the early stages of nodule initiation in the root hairs [47], *RIP1* (*RHIZOBIUM-INDUCED PEROXIDASE1*), *ERN1* (*ERF REQUIRED FOR NODULATION1*), *ENOD11* (*EARLY NODULIN11*), and *NIN1* (*NODULE INCEPTION1*). Gene expression was assessed in 2 cm long root tip segments that were susceptible to rhizobia at 24 h after inoculation with *S. meliloti. RIP1* and *ENOD11* expression were highest in the 3-oxo-C_14_-HSL-exposed roots that had been treated with AB (Figure 6) reflecting the higher numbers of nodules seen later at three weeks post-inoculation (cf. Figure 2). Expression of *ERN1* and *NIN1* showed a similar trend, but while the AHL treatment had a significant effect on their expression, there was no significant interaction with the AB treatment. The results indicated that the combined effect of 3-oxo-C_14_-HSL and the AB treatment on nodule numbers was already reflected in the increased expression of early nodulation genes *RIP1* and *ENOD11* at 24 h after inoculation.

### 2.2. Interactions of the Effects of the AHL 3-oxo-C_14_-HSL and the Root Microbiome

The main aim of this study was to investigate whether the composition of the root colonizing bacterial community of the seedlings altered plant responses to AHLs and also whether the composition of the root microbiome was affected by the application of AHLs. We focused on the AHL 3-oxo-C_14_-HSL because of its positive effect on nodulation. We showed in Figure 1 that the AB treatment was successful in removing the majority of the culturable plant-associated bacteria, which could contribute to AHL production, AHL destruction, or alteration of plant responses to AHLs.

**Figure 7:**
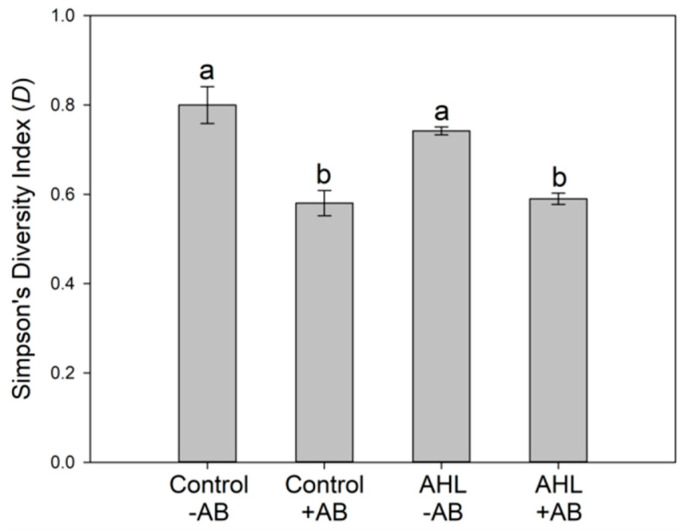
Simpson’s diversity indices of the *Medicago* root-associated bacterial community. Different letters above the bars indicate a statistically significant difference between groups (*p* < 0.05; one-way ANOVA with Tukey’s post hoc test, *n* = 3). AB = antibiotic treatment; AHL = 3-oxo-C_14_-HSL treatment.

We then used 454 pyrosequencing to characterize the composition of bacterial taxa from the roots emerging from seeds treated with or without AB (6 h treatment during seed imbibition) and with and without the AHL 3-oxo-C_14_-HSL (1 μM; exposure over the four days of plant growth). From those treatment groups, we identified 37.7, 15.7, 38, and 19 OTUs (Operational Taxonomic Units) for control – AB, control + AB, AHL − AB, and AHL + AB, respectively (mean of three biological replicates; Table S1). Altogether, 68 different OTUs were identified. Since all seeds were surface-sterilized with sodium hypochlorite before any treatments, and plants were grown on sterile agar plates, the base bacterial diversity was already very low. Simpson’s diversity indices of the *Medicago* root-associated bacterial community showed a further significant reduction of diversity in the AB-treated plants irrespective of AHL treatment (Figure 7).

**Figure 8:**
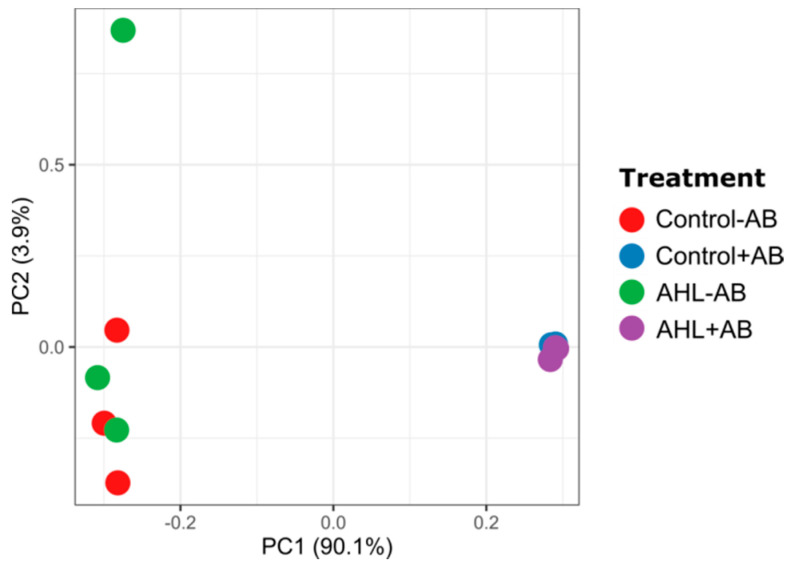
Bacterial community structures of bacteria associated with four-day-old *Medicago* roots with different treatments. Principal component analysis (PCA) showed a difference in the bacterial community structures between the treatments (*n* = 3). AB = antibiotic treatment; AHL = 3-oxo-C_14_-HSL treatment.

Principal component analysis (PCA) further supported the strong effect of the AB treatment in influencing the community structures of the root microbiome (Figure 8).

**Figure 9:**
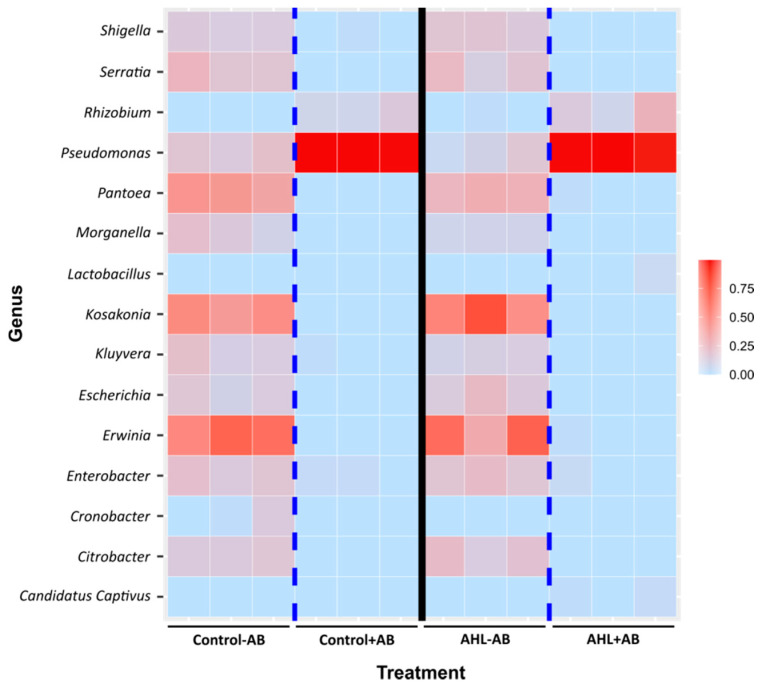
Heatmap of the bacterial genus composition of four-day-old *Medicago* roots with different treatments. The color scale shows the square root of the relative abundance. Each square represents one of the three biological replicates for each treatment. See Table S1 for the list of identified OTUs.

A more detailed comparison of the bacterial composition in the four treatment groups showed a wide range of bacterial diversity in the non-AB treated seedlings, including at least 15 genera. However, antibiotic treatment significantly reduced the number of genera and left only *Pseudomonas* spp. and, at lesser abundance, *Rhizobium* spp. to be identified (Figure 9). None of these *Rhizobium* species included known symbionts of *M. truncatula*. The AHL treatment did not seem to affect the root microbiome at the community structure level (Figure 8) or the genus level (Figure 9) in a major way, as no substantial differences were observed between control − AB and AHL − AB or between control + AB and AHL + AB.

**Figure 10:**
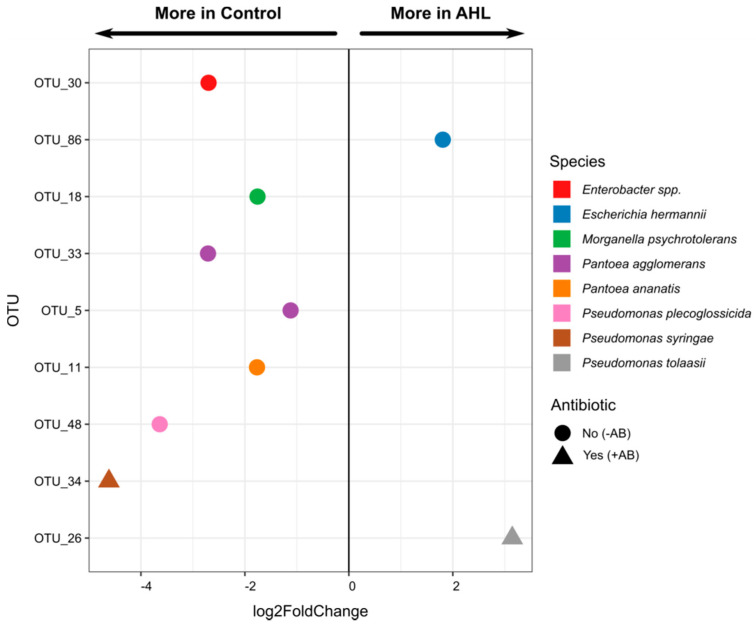
Bacterial OTUs in *Medicago* roots that were significantly different in abundance between the control and AHL (3-oxo-C_14_-HSL)-treated roots (*p* < 0.05). The differential abundances (log2-fold change) of the OTUs between the two groups were calculated with *DESeq2*. The taxonomy of the OTUs is listed in Table S2.

To explore the more detailed effects, *DESeq2* analysis was further performed to elucidate bacterial OTUs that were affected by the AHL treatment. This analysis compared the differential abundance of each OTU between control – AB and AHL – AB and between control + AB and AHL + AB. We found distinct effects of the AHL treatment on –AB compared to +AB-treated roots (Figure 10). Between the control and the AHL treatments, seven and two bacterial OTUs were significantly different in abundance in the −AB and +AB-treated *Medicago* roots, respectively (Figure 10 and Table S2). These included OTUs from *Enterobacter spp*., *Pseudomonas plecoglossicida*, *Pseudomonas syringae*, *Pantoea ananatis, Pantoea agglomerans*, and *Morganella psychrotolerans*, which were more abundant in the control compared to the AHL-treated roots, while two OTUs, classified as *Escherichia hermanii* and *Pseudomonas tolaasii*, were more abundant in AHL-treated roots (Figure 10 and Table S2). Altogether, these results suggested that while the antibiotic treatment had the most severe effect on bacterial abundance and composition, the exposure of plants to AHLs could also alter the composition of plant-associated bacterial communities to some extent. This influence differed between +AB and –AB-treated roots.

## 3. Discussion

The aim of our study was to explore the possibility that the presence and composition of plant-associated bacteria could affect plant responses to AHLs. Many previous studies have demonstrated that exposure of plants to bacterial AHLs affects diverse phenotypes, including plant growth, development, and immune responses [13,16,19,20,22,23,25,26,27,48]. These studies typically grow plants under semi-sterile conditions with surface-sterilized seeds to minimize the influence of AHLs from contaminating bacteria. We showed here that 1) even roots growing from surface-sterilized seeds of field-grown *M. truncatula* that did not show any obvious signs of contamination under plate-grown conditions contained multiple bacterial species, and 2) that the presence of these plant-associated bacteria could modulate the response of the plant to AHLs. We further showed that the interaction between the AHL effect and the bacterial community was dependent on the AHL structure and the phenotype investigated.

In our experiments, we used an antibiotic treatment that was applied for 6 h immediately after surface-sterilization of the seeds with sodium hypochlorite. We did not find any systematic effects of the antibiotic treatment on plant phenotypes, and the antibiotic was removed by extensive washing; therefore, we believed that it is unlikely that the influence of the antibiotic was a direct effect on the plant, but that it acted by removing the majority of bacterial taxa present in the plants. Because the seeds we used were field-grown, it is likely that the origin of some of the bacteria is located either on or below the seed coat, but some of the bacteria might also be endophytic. We did not further investigate the location or origin of these bacteria, but simply concluded for the purpose of this study that they were associated with the plant. The bacteria that we found associated with *M. truncatula* seedlings belonged to genera that have been found associated with plants in other studies. The most abundant genera were *Erwinia*, a genus with many known plant pathogens [49], *Enterobacter,* some of which are plant growth-promoting endophytes [50,51], and *Pantoea* species, which include seed endophytes and plant growth-promoting species as well as pathogens [52,53]. Other genera represented were species of *Rhizobium*, *Pseudomonas,* and *Serratia*. Similarly, identification of *Medicago sativa* seed endophytes (from surface-sterilized seeds) also found members of *Enterobacter*, *Pantoea*, non-nodulating rhizobia, and *Pseudomonas* species, among others [54]. A recent microbiome study of *M. truncatula* found various species of *Pseudomonas* and *Rhizobium* species associated with *M. truncatula* roots, although these were from soil-grown plants [55]. While in that microbiome study, Proteobacteria represented more than 50% of root-associated taxa, we found Proteobacteria almost exclusively in our study, suggesting that the seed treatment could have selectively removed several taxa or that soil exposure increased members of the other taxa. Future experiments could be aimed at a more detailed metagenomics analysis of the bacterial species inhabiting the field-grown *M. truncatula* seeds. Treatment with the antibiotics reduced total numbers and diversity of bacteria drastically and shifted the relative abundance of bacteria strongly towards *Pseudomonas* species, with lower numbers of *Rhizobium* species, although none of these were known symbionts of *M. truncatula*. The predominance of *Pseudomonas* spp. in antibiotic-treated roots is likely a result of *Pseudomonas sp*. being able to develop antibiotic resistance to a broad range of antibiotics [56]. While we only used one broad-spectrum antibiotic in this study, it is likely that other methods of manipulating the *M. truncatula* microbiome could have yielded different results.

The shift of bacterial community composition and abundance associated with *M. truncatula* clearly affected the responses of the roots to AHLs. This could occur via several mechanisms, including (1) background production of AHLs by the plant-associated bacteria that are perceived by the plant at the same time as the AHL used as the experimental treatment, (2) destruction of the AHL used as the treatment by plant-associated bacteria, or (3) modulation of plant responses by plant-associated bacteria independent of their AHL production. These three mechanisms are further discussed below.

The predominant genera—*Erwinia*, *Enterobacter*, *Pantoea*, *Pseudomonas,* and *Rhizobium—*are all known to synthesize AHLs, although of different structures. Some of the AHLs synthesized by these species overlap the AHLs used here, for example, *S. meliloti* produces C_8_-HSL, 3-oxo-C_8_-HSL, C_14_-HSL, 3-oxo-C_14_-HSL, and C_18_-HSL [14,46], while *Pantoea* spp. [57] and *Erwinia* spp. [58] synthesize 3-oxo-C_8_-HSL and *Pseudomonas* spp. [7] and *Enterobacter* spp. [59] synthesize C_4_-HSL. However, the exact AHL structures of the specific species of most of the identified bacteria in our study are not known. Thus, the perception of external AHLs by the plant, if colonized by these bacteria, is likely a mixed response to the combination of AHLs the plant perceives. For example, 3-oxo-C_14_-HSL appears to enhance nodulation in the absence of substantial numbers of plant-associated bacteria and thus likely in the absence of substantial concentrations of other AHLs. However, other AHLs may have negative effects on nodule numbers. Thus, if the plant perceives a mixture of these AHLs, the net outcome might be unchanged numbers of nodules. This would depend, of course, on the location where these AHLs are made and perceived, the mechanism by which different AHLs are perceived, and the mechanisms by which each of them acts. Currently, no plant receptors that directly bind different AHLs have been identified.

Another important consideration is whether the bacteria identified to colonize *M. truncatula* roots would actually occur at densities at which effective concentrations of AHLs are produced. We estimated the number of culturable bacteria recovered from the non-antibiotic-treated roots to be around 120,000 per root, approximately 4 cm long. This does not take into account that there would be unculturable bacteria and that different members of those microbial populations would likely to be dense in some locations and absent from other parts of the root. Estimations of quorum sizes for bacteria on dry leaf surfaces, where diffusion is limited, is small, with populations as low as 10 cells able to communicate via AHLs, while this number increases in wetter environments with higher diffusion rates [60]. The reporter used to detect AHLs start to respond to 3-oxo-C_6_-HSL at 100 nM [60]. Similar results have been reported by microscopic analysis of quorum sensing calling distances on root surfaces of sand-grown tomato plants, uncovering that AHL-medicated quorum sensing occurs even at low bacterial densities and can be exerted over distances far beyond the length of a bacterial cell [61]. This has been done using an AHL reporter sensitive to concentrations above 20 nM of 3-oxo-C_12_-HSL. These studies suggest that even small clumps of cells on a plant surface could contain locally significant concentrations of AHLs. Whether these are sufficient to trigger plant responses would be interesting to investigate.

Secondly, some of the identified plant-associated bacteria are able to destroy AHLs, often from other species, and this quorum quenching mechanism is likely a widespread mechanism for interfering with quorum sensing across bacterial species [62,63]. Quorum quenching activities have been characterized in rhizobia [63] and *Pseudomonas* [64] species, but could also be present in some of the other species identified here. Thus, the presence of plant-associated bacteria could alter the active concentrations of any applied AHLs, lessening or altering their effect on the plant.

Thirdly, the presence of plant-associated bacteria could potentially induce a number of biochemical and physiological responses in the plant that indirectly interfere with the response to an applied AHL. Plant-associated bacteria are able to synthesize hormones, effectors, and signals that could interfere with plant responses to AHLs, in particular, those that alter development and defense [65]. We observed significant effects of the antibiotic treatment alone in some of the AHL treatments, for example, on plant biomass (Figure 4). This might reflect the production of plant hormones by the plant-associated bacteria, although this would have to be investigated in detail by further experiments. If plant hormone responses or plant immune responses were altered by the ‘background’ presence of plant-associated bacteria, the AHLs that cause changes to the same pathways might lead to different overall responses by the plant. Some of the plant-associated microbes that were reduced by the antibiotic treatment included known plant pathogens, including *Erwinia*, *Pantoea,* and *Serratia* spp., although their direct effects on *M. truncatula* were not studied in detail.

The focus of our study was mainly on nodulation, as rhizobia are well documented to require AHL signaling for effective symbiosis [35]. We confirmed that the application of 3-oxo-C_14_-HSL at 1 μM concentration significantly increased nodule numbers, similar to what previous studies found [41,42], but only in antibiotic-treated roots. While roots in our previous study [41] were treated with the same antibiotic, a separate study, which found a significant but relatively smaller increase in nodule number than shown here, did not treat seeds with antibiotics, and the source of seeds and growing conditions were different [42]. It would be interesting to compare the effects of AHLs on different batches of seeds of the same species originating from different sources with different inherent seed-associated bacteria side by side in the same experiment. None of the other AHLs had positive effects on nodule numbers in this study, but some had effects in another study [42]. This highlights the difficulty of comparison between studies, with some of the variability likely due to the presence of seed-borne bacteria and further variability likely due to growth conditions used. It is also likely that different strains of rhizobia could exert different effects. The *S. meliloti* 1021 strain used here lacks one of the AHL receptors, ExpR, which affects some of its AHL-dependent behaviors [66]. It would, therefore, be interesting to test the nodulation responses in strains encoding a functional copy of *ExpR*. It would, furthermore, be interesting to examine the effects of AHLs on nitrogen fixation, which we did not do here. At the time of harvesting at three weeks after inoculation, biomass changes accompanying the higher nodule numbers in 3-oxo-C_14_-HSL-treated roots were not evident, suggesting that any potential increases in nitrogen fixation had not occurred or not led to enhanced biomass (yet), but this would have to be quantified in future studies.

We also showed that the effect of 3-oxo-C_14_-HSL was manifested in the increased expression of early nodulation genes within the first 24 h after inoculation with rhizobia. This suggested that this AHL, which is produced by its symbiont *S. meliloti* [14], was specifically perceived by the plant host and enabled different responses to rhizobial Nod factors. The early nodulation genes—*ENOD11*, *ERN1*, *NIN1,* and *RIP1—*which showed the highest expression in 3-oxo-C_14_-HSL-treated roots in the presence of the antibiotics, when most nodules were observed, are inducible by Nod factors in *M. truncatula* root hairs within 24 h and are known markers for successful Nod factor signaling [47]. However, only *RIP1* and *ENOD11* expression levels showed a similar significant interaction between AHL and antibiotic treatment, as was seen for nodule numbers (Figure 6). RIP1 is a Rhizobium-induced peroxidase necessary for the formation of reactive oxygen species that are required for the infection of rhizobia [67], while ENOD11 is a repetitive proline-rich cell-wall protein that is an early marker for successful infection [68]. This result suggested that 3-oxo-C_14_-HSL acted at the earliest stages of nodulation. In the future, it will be interesting to investigate the mechanism of 3-oxo-C_14_-HSL perception and action during nodulation in *M. truncatula* in more detail, for example, through quantifying infection events and more comprehensive analysis of gene expression changes, including defense genes that could regulate early infection events. Interestingly, the same AHL also affects immune responses in non-legumes [26,27]. An additional interesting aspect to examine would be to test whether 3-oxo-C_14_-HSL may affect Ca^2+^-signaling in the root hair since Ca^2+^ spiking is one of the earliest responses to Nod factors from rhizobia [69] and the short-chain AHL—C_4_-HSL—has been shown to trigger changes in Ca^2+^ signaling in Arabidopsis [70].

Apart from the specific nodulation responses to AHLs, there were negligible effects on root length, lateral root numbers, and plant biomass. Other studies have found similar results at 1 μM concentrations of AHLs, while other concentrations can have strong effects on root elongation [16,18,22,41,42,71]. This underlines the importance of interpreting AHL responses in the context of their concentrations.

Overall, our finding that some AHL responses were dependent on the presence of plant-associated bacteria clearly cautions studies on plant responses to AHLs because it is unlikely that results obtained under sterile or semi-sterile laboratory conditions can be translated to plants growing in the field, where plants are exposed to many different AHLs at once, as well as a myriad of other signals from plant-associated bacteria. It might also explain why different studies do not always find similar results or why different plant species show different responses to the same AHLs [72].

Finally, an unexpected result from our study was that the application of 3-oxo-C_14_-HSL to *M. truncatula* altered the composition of the plant-associated bacterial community. This was evident both in the presence and absence of antibiotics. While this effect was not large, several bacterial species were significantly affected in abundance by AHL treatment. This suggested a further complication of studies looking at AHL responses in plants (or other eukaryotes), as the effect of an applied AHL could include indirect effects of bacteria associated with the host. We currently do not understand the mechanism by which AHLs could shift bacterial community composition associated with the plant host. AHLs might alter the exudation of secondary metabolites that could affect bacterial community composition. For example, exposure of *M. truncatula* to AHLs has been shown to alter the expression of the flavonoid synthesis pathway [13] and the accumulation of flavonoid metabolites [41], and flavonoids can alter microbiome composition [73]. In addition, exposure of *M. truncatula* to AHLs has altered the exudation of metabolites that can interfere with quorum sensing between bacteria [13]. Thus, altered quorum sensing in the plant-associated microbiome could alter its community composition through alteration of bacterial movement, selective biofilm formation, competition for nutrients, or plasmid transfer, which are regulated by quorum sensing in rhizobia [35]. Altogether, a complex picture emerges that warrants further investigation between plant hosts, their associated microbiome, and the role of bacterial quorum sensing signals in shaping both plant phenotypes and the microbial community composition.

Our study could be extended in the future in many directions. First, while the choice of antibiotics and the treatments applied were likely to influence the outcome of our study, our study demonstrated that, in principle, a shift in the plant-associated microbiome did make a difference in the outcome of plant AHL responses. Further studies could be aimed at varying the way the microbiome is altered. For example, rather than removing existing taxa, the addition of specific species of bacteria to the plant could be trialed. Additional experiments should also be aimed at gaining a comprehensive view of the actual AHL structures and concentrations present in and around roots over time and in different locations. AHL concentrations are likely to be very dynamic in time and often variable, depending on the location different bacteria are present in or around the root. In addition, the AHLs were mixed into the growth media that the plants were growing on and would likely have started breaking down during the experiment [74], although the pH of the medium was kept acidic. It is hard to predict what the actual active concentrations of AHLs were at different times and whether breakdown products might have altered the responses over time. Our study did not capture that complexity, and this could be explored in the future. The fact that the much shorter-term exposure to AHLs caused changes in nodulin gene expression in a similar pattern to the increased nodule numbers three weeks later suggested that the effect of 3-oxo-C_14_-HSL was quite specific.

## 4. Materials and Methods

### 4.1. Media and Plant Growth Conditions

Seeds of *M. truncatula* Jemalong A17 were purchased from the South Australian Research and Development Institute in Adelaide. These seeds had been harvested from field-grown plants. Seeds were scarified with sandpaper, surface-sterilized for 10 min in 6% (w/v) sodium hypochlorite, followed by five washes with sterile water. Seeds were then either soaked for 6 h in a solution containing 200 mg L^−1^ amoxicillin and 50 mg L^−1^ clavulanic acid (Sigma Aldrich, North Ryde, Australia; antibiotic treatment, +AB) or soaked in sterile water (control, –AB), in both cases on a rotating shaker. After this, seeds were rinsed by five washes with sterile water to remove the antibiotics and placed at 4 °C for 48 h on Fåhraeus medium [75] agar plates adjusted to pH 6.5 (unbuffered). The pH of the medium further acidified, likely from root exudates, within two days of placing *M. truncatula* plants onto the plates, and was reduced to ~pH 5 within two days and ~pH 4.5 after three weeks. An acidic pH was important to minimize the alkaline hydrolysis of the lactone ring of the AHLs [74,76]. Seeds were then germinated at 25 °C for 16 h in darkness.

The AHLs were purchased as the naturally occurring L-forms from Cayman Chemicals (Ann Arbor, Michigan, USA), dissolved in dimethyl sulfoxide (DMSO, Sigma Aldrich, North Ryde, Australia; stock solution 70 mM), filter-sterilized, and diluted to a final concentration of 1 μM into the appropriate plant growth medium following autoclaving and cooling of the medium. Solvent diluted to the same concentration as used for AHLs was used as a negative control. The AHL concentration was chosen because we previously found it to affect nodulation in *M. truncatula* [41].

Ten *Medicago* seedlings with uniform root length (approximately 1 cm) were transferred to square Petri dishes (245 mm × 245 mm × 18 mm) (Corning, New York, NY, USA) containing Fåhraeus medium (pH 6.5) with the AHL (AHL treatment containing DMSO at 1:70,000 final concentration) or the DMSO solvent (control treatment with DMSO solvent; 1:70,000 final concentration). Each treatment was replicated three times (i.e., three plates with ten seedlings each), and the plates were incubated in a growth room in a randomized block design, with the controlled temperature at 25 °C, 120 μmol m^−2^ s^−1^ of light intensity, and 16/8 h day/night cycle. Three days after the transfer, the root tips of *Medicago* seedlings were then inoculated with 10 μL *Sinorhizobium meliloti* strain 1021 in a liquid culture grown in Bergersen’s modified medium [77] to an OD_600_ of 0.1. Plants were grown for another 21 days after inoculation and harvested. Nodules were counted under a stereomicroscope. All plates were then scanned with a flatbed scanner (Epson Perfection V700 Photo, Epson, North Ryde, Australia) at 600 dpi, and the main root length was measured from the image using ImageJ. For the determination of plant dry biomass, three biological replicates with 10 plants each per treatment were placed into a paper envelope and dried at 60 °C for 72 h in an incubator (Axyos, Brendale, Australia). Samples were carefully handled in an airtight plastic box containing silica gel type III (Sigma Aldrich, North Ryde, Australia) to avoid moisture in the samples while they were weighed. Total dry biomass was measured on balance (Mettler Toledo, Greifensee, Switzerland).

### 4.2. Quantitative Reverse Transcription-Polymerase Chain Reaction (qRT-PCR)

Two-centimeter-long root tip segments were harvested from five *M. truncatula* seedlings for each biological replicate. Three biological replicates were used for each treatment. Samples were finely ground in liquid nitrogen using mortars and pestles. Approximately 60 mg of finely ground tissue was transferred to a new Eppendorf tube for RNA extraction using the Spectrum Plant Total RNA kit (Sigma Aldrich, North Ryde, Australia). The extracted and purified RNA was quantified on a NanoDrop ND1000 UV/Vis spectrophotometer (Thermo Fisher Scientific, Scoresby, Australia). cDNA was synthesized from equal amounts of RNA for all treatments using the Superscript III First Strand cDNA Synthesis kit (Thermo Fisher Scientific, Scoresby, Australia).

qRT-PCR primers (Table 1) were designed using Primer3Plus [78], Quantprime [79], and ProbeFinder software in the Universal Probe Library Assay Design Center (Roche Applied Science, Penzberg, Germany). Different parameters were taken into consideration, such as melting temperature (Tm) between 58 °C and 61 °C, primer size no more than 23 base pairs, PCR amplicon lengths of 100–150 base pairs (bp), and limited self-complimentary. The specificity of the transcripts was tested on *M. truncatula* cDNA dilutions (1/10, 1/100, and 1/1000). *GAPDH* (glyceraldehyde 3-phosphate dehydrogenase) was chosen as the most stably expressed housekeeping gene, and all ratios presented in relation to *GAPDH*. The amplification was done on an ABI 7900HT Sequence Detection System (Applied Biosystems, Foster City, CA, USA) using SYBRGreen (Applied Biosystems, Foster City, CA, USA). The cycle program used was as follows: Initial denaturation at 95 °C for 20 s, followed by 40 cycles at 95 °C (1 sec) and 60 °C (20 sec). A melt curve was established using 95 °C for 15 s, 60 °C for 1 min, followed by 95 °C for 15 s.

The qRT-PCR data analysis was done according to [81] using the software LinRegPCR version 2014.2 [82]. Statistical analysis was carried out with GraphPad Prism (Graph Pad, San Diego, CA, USA) version 5.02 using one-way ANOVA on three biological replicates (three technical replicates for each biological replicate were done).

### 4.3. Estimation of Bacteria From M. truncatula Roots by Culturing and 16S Amplification

*Medicago* roots of four-day-old seedlings, derived from seeds treated with (+AB) and without antibiotics (−AB), and either grown on Fåhraeus media containing 1 μM 3-oxo-C_14_-HSL or a solvent control, were weighed in 1.5 mL Eppendorf tubes on balance (Mettler Toledo, Greifensee, Switzerland). Roots were ground with autoclaved glass powder using a sterile (autoclaved) mortar and pestle. One milliliter of sterile phosphate-buffered saline (PBS; 137 mM NaCl, 2.7 mM KCl, 10 mM Na_2_HPO_4_, 1.8 mM KH_2_PO_4_) was added to the sample, and the bacteria were extracted by vortexing for 15 s. Serial dilutions were made with sterile PBS, followed by plating on LB agar [83] and R2A agar (Difco, Thermo Fisher Scientific, Scoresby, Australia; yeast extract 0.5 g/L, proteose peptone No. 3 0.5 g/L, casamino acids 0.5g/L, dextrose 0.5 g/L, soluble starch 0.5 g/L, sodium pyruvate 0.3 g/L, dipotassium phosphate 0.3g/L, magnesium sulfate 0.05 g/L, agar 15 g/L). Plates were incubated in the dark at 28 °C for seven days, and colonies were counted by eye. The presented results are the averages of colonies grown on both media.

### 4.4. Bacterial Community Analysis

To examine the effects of the antibiotics and the AHL treatment (1 μM 3-oxo-C_14_-HSL) on the root colonizing bacterial community, *Medicago* seeds were surface sterilized with 6% (w/v) sodium hypochlorite and treated with (+AB) and without (−AB) the antibiotics, as described above. The seeds were grown on control (with DMSO solvent; 1:70,000 final concentration) or AHL (with 1 μM 3-oxo-C_14_-HSL, also containing 1:70,000 dilution of DMSO)-containing Fåhraeus agar plates. A total of four treatments (control – AB, control + AB, AHL – AB, and AHL + AB) were prepared, with three biological replicates per treatment, and five plants per replicate. Roots were collected four days after germination for DNA extraction. We chose this time point because it was the same as the time point used to analyze gene expression changes in response to AHLs and antibiotic treatments, as described above.

DNA was extracted from the *Medicago* root samples with the DNeasy^®^ Plant Mini kit (QIAGEN^®^, Germantown, MD, USA) following the manufacturer’s instructions. Bacterial 16S ribosomal RNA (rRNA) genes were PCR amplified with 799F (5′-AAC MGG ATT AGA TAC CCK G-3′; [84]) and 1193R (5′-ACG TCA TCC CCA CCT TCC-3′; [85]) primer set. The PCR was conducted with Platinum^®^Taq DNA Polymerase (Invitrogen, Carlsbad, CA, USA) and run in a C1000 Touch™ Thermal Cycler (Biorad, Hercules, CA, USA). Conditions were as follows: initial denaturation at 95 °C for 3 min, followed by 35 cycles of 95 °C (30 s), 55 °C (30 s), 72 °C (1 min), with the final elongation step at 72 °C for 5 min. The PCR products were then separated on a 1.2% agarose gel at 100 volts for 30–45 min to separate the bacterial 16S rRNA gene amplicons (~400 bp) from the plant mitochondrial 18S rRNA gene amplicon (~800 bp). The bacterial bands were excised from the gel, and the PCR products recovered and purified with the QIAquick Gel Extraction kit (QIAGEN^®^, Germantown, MD, USA). Barcoded amplicon sequencing processes were performed by Molecular Research LP (Shallowater, Texas, USA) under the trademark service (bTEFAP^®^) described by [86,87]. Samples were sequenced with Roche 454 FLX titanium instruments and reagents, following manufacturer’s guidelines. For the sequencing data processing, initially, the barcodes and primers were removed from the sequences. Short sequences (<200 bp), sequences with ambiguous base calls, and sequences with homopolymer runs exceeding 6 bp were discarded. Sequences were denoised, and operational taxonomic units (OTUs) were defined at 97% similarity, followed by the removal of singleton sequences and chimeras [86,87,88,89,90,91]. Taxonomies were assigned to the OTUs using BLASTn against a curated database derived from Greengenes [92], Ribosomal Database Project (RDP) [93], and the NCBI Taxonomy database [94].

### 4.5. Analysis of Microbiome Data

From the valid sequences, an equal number of sequences (1420) was rarefied from each sample to generate an OTU table (Table S1). The OTU table was used for subsequent statistical analyses. Most of the statistical analyses were performed in the R v3.6.3 environment [95]. For visualization of the community relationships between the sample groups, Hellinger transformation was applied to the OTU table [96], and principal component analysis (PCA) and Simpson’s diversity indices (*D*) were calculated with *vegan* package [97]. OTUs with differential abundance between two sample groups (control and AHL) were identified with the *DESeq2* package [98] by converting the data with the *phyloseq* package [99], as described by [100].

## 5. Conclusions

Our results showed that plant phenotypic responses to bacterial AHLs could be modified by the abundance and composition of plant-associated bacterial communities. In addition, the modification of the AHL responses was specific for the AHL-tested and the phenotype studied. This indicated that studies examining the responses of plants to AHLs need to carefully consider the presence of plant-associated microbes that may directly or indirectly alter plant responses to AHLs. Interestingly, exposure of plants to AHLs also altered the relative abundance of specific bacterial taxa associated with the plant. Future studies could be aimed at determining the molecular mechanisms by which AHLs, plants, and associated microbes interact, for example, through molecular mimicry, quorum quenching, or indirect changes to plant metabolism and defense responses.

## Figures and Tables

**Table 1:** The qRT-PCR primers used for assessing the expression of nodulation genes.

Gene Name	Primer Sequence	Reference
*GAPDHF*	5′ TGCCTACCGTCGATGTTTCAGT 3′	[80]
*GAPDHR*	5′ TTGCCCTCTGATTCCTCCTTG 3′	[80]
*ERN1F*	5′ TGCATGCCTTCTTCGAGGTTCG 3′	This study
*ERN1R*	5′ TCCTGGAAGCAAGAGGAGAATCC 3′	This study
*RIP1F*	5′ GCTAGATGATACCCCAAATTTCA 3′	This study
*RIP1R*	5′ CCACAGAAAATCCTCTGATTGA 3′	This study
*MtNIN1F*	5′ GGGAGAAAGTCCGGGGACAA 3′	[78]
*MtNIN1R*	5′ GACACACACCGATGCTCTTTGC 3′	[78]
*ENOD11F*	5′ TATGGTAACCAGCCTCCACCTAGC 3′	This study
*ENOD11R*	5′ GCATTGGTAAACCTTGTTGCTTGC 3′	This study

## Supplementary Materials

The following are available online at https://www.mdpi.com/2223-7747/9/6/777/s1, Table S1: OTUs identified from bacterial communities associated with *M. truncatula* in the absence and presence of antibiotics and 3-oxo-C_14_-HSL. Table S2: Taxonomy of the OTUs affected by the presence of AHLs.
